# Associations Between Nasal Receptors and Olfactory Dysfunction and Dysgeusia in Coronavirus Disease 2019 (COVID-19)

**DOI:** 10.3390/jcm15041659

**Published:** 2026-02-22

**Authors:** Ana María Piqueras-Sánchez, José Francisco López-Gil, Diego Hellín-Meseguer, Juan Cabezas-Herrera, Ginés Francisco Blesa-Llaona, José Meseguer-Cabezas, Enrique Bernal-Morell, Alfredo Minguela-Puras, José Antonio Díaz-Manzano

**Affiliations:** 1 Department of Otolaryngology, Virgen de la Arrixaca University Clinical Hospital (HCUVA), 30120 Murcia, Spain; diego.hellin@um.es (D.H.-M.); gblesa@gmail.com (G.F.B.-L.); pepechinatto@gmail.com (J.M.-C.); jdiazmanzano@hotmail.com (J.A.D.-M.); 2 Department of Otolaryngology, Faculty of Medicine, Campus de Ciencias de la Salud, University of Murcia (UMU), 30100 Murcia, Spain; 3 School of Medicine, Universidad Espíritu Santo, Samborondón 092301, Ecuador; 4 Faculty of Health Sciences, Universidad Autónoma de Chile, Santiago 7500000, Chile; 5 Biomedical Research Institute of Murcia (IMIB), 30120 Murcia, Spain; jcabezas@um.es (J.C.-H.); ebm.hgurs@gmail.com (E.B.-M.); alfredo.minguela@carm.es (A.M.-P.); 6 Molecular Therapy and Biomarkers Research Group, Virgen de la Arrixaca University Clinical Hospital (HCUVA), 30120 Murcia, Spain; 7 Department of Internal Medicine, Section of Infectious Diseases, Reina Sofía University Hospital (HRSU), University of Murcia (UMU), 30003 Murcia, Spain; 8 Immunology Service, Virgen de la Arrixaca University Clinical Hospital (HCUVA), 30120 Murcia, Spain

**Keywords:** COVID-19, SARS-CoV-2, ACE2, TMPRSS2, furin, neuropilin-1, nasal epithelium, olfactory dysfunction, dysgeusia

## Abstract

**Background/Objectives:** Olfactory dysfunction and dysgeusia are common neurosensory manifestations of Coronavirus Disease 2019 (COVID-19), affecting approximately 60% of patients. These symptoms have been mechanistically linked to receptors involved in Severe Acute Respiratory Syndrome Coronavirus 2 (SARS-CoV-2) cell entry, including angiotensin-converting enzyme 2 (ACE2), transmembrane protease serine 2 (TMPRSS2), furin, and neuropilin-1 (NRP1), which are highly expressed in the olfactory epithelium. Nevertheless, clinical evidence supporting a direct association between receptor expression and sensory impairment remains inconsistent. **Methods:** We conducted a multicenter, observational, cross-sectional study including 104 adults with polymerase chain reaction–confirmed SARS-CoV-2 infection during the first and second pandemic waves. Approximately 75 days after diagnosis, nasal and/or pharyngeal samples were obtained to quantify gene expression levels of ACE2, TMPRSS2, furin, and NRP1 using quantitative polymerase chain reaction. Olfactory dysfunction and dysgeusia were recorded as dichotomous variables. Logistic regression analyses were performed with adjustment for age, sex, and race, considering receptor expression as continuous variables and as tertiles. Missing data were addressed using multiple imputation methods. **Results:** Olfactory dysfunction was reported by 37.5% of participants, and dysgeusia by 36.5%. No statistically significant associations were observed between baseline expression levels of ACE2, TMPRSS2, furin, or NRP1 and the presence of olfactory dysfunction or dysgeusia in either adjusted continuous or categorical models. Although these associations did not reach statistical significance, higher ACE2 and furin expression showed a nonsignificant trend toward an increased probability of sensory alterations, whereas intermediate NRP1 levels were associated with lower disease severity. **Conclusions:** COVID-19-related olfactory dysfunction and dysgeusia do not appear to be directly determined by isolated baseline expression of SARS-CoV-2 entry receptors. These findings support a multifactorial and dynamic pathophysiological model involving temporal receptor regulation, inflammatory processes, and host-related factors, highlighting the need for longitudinal and interventional studies.

## 1. Introduction

Coronavirus Disease 2019 (COVID-19), caused by the Severe Acute Respiratory Syndrome Coronavirus 2 Respiratory Virus (SARS-CoV-2), ranges from low-mild respiratory symptoms to severe systemic complications. Among the neurosensory symptoms observed are alterations in smell and taste, whose prevalence has been estimated at approximately 60.1% of patients [1]. Olfactory dysfunction associated with COVID-19 presents an epidemiological pattern, with a higher incidence in women and young individuals and a lower frequency in patients requiring hospitalization [2,3]. However, the clinical relevance and prevalence of this disorder have varied throughout the pandemic [4,5,6,7].

From a pathophysiological perspective, the literature describes high expression of angiotensin-converting enzyme 2 (ACE2), transmembrane serine protease (TMPRSS2), and furin in the olfactory epithelium, suggesting their possible involvement in viral entry and the development of olfactory dysfunction [8,9,10,11,12]. However, recent evidence suggests that neuropilin-1 (NRP1) could play a key role, given its high expression in the olfactory neuroepithelium and its ability to facilitate the binding and entry of SARS-CoV-2 into neuro-olfactory cells [9,10]. Findings from postmortem studies reinforce this hypothesis by demonstrating abundant expression of NRP1 in patients who died from COVID-19 [4,11,13].

SARS-CoV-2-induced olfactory impairment has also been proposed as a possible prognostic marker of this disease, as its presence has been associated with milder clinical forms [4,11,13]. Two main pathophysiological mechanisms are postulated: inflammatory, secondary to edema and inflammation of the nasal mucosa, and neuronal, due to the direct involvement of the olfactory neuroepithelium and olfactory nerve. The distribution of ACE2 in the central nervous system (CNS) supports the hypothesis of direct or indirect viral invasion, as well as the possibility of retrograde axonal transport, which contributes to neural inflammation and loss of smell [11,14,15].

Clinically, olfactory dysfunction can be divided into qualitative alterations, such as phantosmia and cacosmia, and quantitative alterations, among which hyposmia and anosmia predominate. The latter is the most common presentation and, in most cases, is not accompanied by other otolaryngological symptoms and may even manifest as the first or only symptom (11% of cases) [2,11]. The duration of this symptom varies in the literature; although SARS-CoV-2 is associated with faster recovery than other respiratory viruses are [4,11,16], a highly variable percentage of the literature (5–50%) reports persistent symptoms beyond 4–12 weeks [1,4].

Olfactory function can be assessed via subjective and objective tests, with the latter being more sensitive in detecting abnormalities. Among these, functional magnetic resonance imaging stands out, although its routine use has not yet been established [17,18]. Currently, there is no specific effective therapy for this condition [4]. Although intranasal corticosteroid therapy has shown controversial results [19,20,21,22], olfactory training is emerging as a promising strategy, with improvements in olfactory thresholds and the ability to identify and discriminate odors [4].

Accordingly, this study was designed to explore, in an associative and non-causal manner, whether the expression levels of SARS-CoV-2 entry-related genes measured in nasal and oropharyngeal swab-derived samples during the post-acute phase of infection are associated with the presence or absence of olfactory and/or gustatory dysfunction.

## 2. Materials and Methods

**Study design and population**

An observational, analytical, cross-sectional, multicenter study was conducted to evaluate the molecular expression of the ACE2, TMPRSS2, furin, and NRP1 receptors in nasal and/or pharyngeal mucosal tissues and compare them with the presence of olfactory dysfunction and dysgeusia. The study included patients who contracted COVID-19 during the first and second phases of the pandemic, all of whom had a confirmed diagnosis via polymerase chain reaction (PCR). The first phase was dominated by the ancestral SARS-CoV-2 strain, whereas the second phase was largely associated with variants harboring the D614G mutation, with the Alpha variant (B.1.1.7) emerging toward the end of this period, according to contemporaneous epidemiological data.

From an original pool of 201 adults with laboratory-confirmed SARS-CoV-2 infection, 104 patients ultimately met the criteria for inclusion in the analysis. Subjects who were not included in the final cohort either declined to participate in the study or were excluded because of issues related to sample collection.

Olfactory and gustatory dysfunction were assessed at the post-acute study visit, approximately 75 days after confirmed SARS-CoV-2 infection. At this visit, participants were asked whether they had experienced olfactory and/or gustatory dysfunction at any time during their COVID-19 illness. Responses were recorded as a binary variable (present/absent). The aim of this assessment was not to characterize the clinical features, severity, timing, or evolution of these symptoms, but rather to determine their occurrence in the context of SARS-CoV-2 infection and to explore their association with nasal expression levels of SARS-CoV-2 entry-related genes. Detailed information regarding symptom onset, duration, or progression during the acute phase was not collected, as this was beyond the scope of the study.

Nasal and oropharyngeal swab samples were obtained in a post-acute phase of the infection from all participants and analyzed separately to determine receptor expression. Only one participant was collected by oropharyngeal swab due to refusal to undergo nasal sampling. This sample was processed using the same protocol and is explicitly acknowledged as an exception.

**Procedures**

Clinical samples were collected via nasal or oropharyngeal swabs. Following collection, the samples underwent a standardized processing workflow that included centrifugation, complementary DNA synthesis, and quantitative amplification techniques to assess receptor-related gene expression. The expression levels were adjusted relative to those of glyceraldehyde-3-phosphate dehydrogenase (GAPDH), which served as the endogenous control.

This study incorporated the assessment of the following domains:

- The expression of ACE2, TMPRSS2, furin, and NRP1 in nasal and/or pharyngeal epithelial tissues were quantified. Total RNA was isolated via the InviTrap© Spin Universal RNA Mini Kit (STRATEC Molecular GmbH, Berlin, Germany), and downstream expression analysis was performed via the GeneAmp RNA PCR Core Kit (Applied Biosystems, Foster City, CA, USA; N808-0143).
- Participant demographic characteristics, including age, biological sex, and self-identified race, were documented.
- The presence or absence of anosmia and dysgeusia was recorded as a binary clinical variable.

**Statistical analysis**

The distributional properties of the study variables were examined via graphical methods, including quantile-quantile plots and kernel density curves, in conjunction with the Shapiro-Wilk test. Given the predominance of non-normal distributions, summary statistics are presented as medians and interquartile ranges (IQRs). The relationship between receptor expression levels and olfactory dysfunction and dysgeusia was evaluated via generalized linear modeling, which assumes a binomial error structure. All models incorporated adjustments for potential confounders, specifically age, sex, and race. Receptor expression in nasal tissue was modeled as a continuous measure, scaled per 100-unit increment, and as an ordinal categorical variable based on tertile classification (low, medium, high), allowing the exploration of possible nonlinear effects. Effect estimates are reported as odds ratios (ORs) accompanied by 95% confidence intervals (CIs). Incomplete observations were addressed via a multiple imputation strategy implemented via the ‘mice’ package. Statistical significance was set as *p* < 0.05. All analyses were conducted via RStudio (Posit, version 2024.04.1+748; Vienna, Austria).

## 3. Results

The demographic characteristics of the participants are summarized in Table 1. The study population was predominantly male, with 69 men (66.3%) and 35 women (33.7%), and the median age was 58.5 years (IQR: 16.2). Most participants self-identified as Caucasian, accounting for 81.7% of the sample (*n* = 85). Furin expression showed a median of 212.3 units (IQR 105.1) when all observations, including missing values, were considered and 213.0 units (IQR 103.8) after the exclusion of incomplete data. The median ACE2 expression was 2098.3 units, with an interquartile range of 1271.3. For NRP1, the median level reached 1904.3 units (IQR 1627.6) in analyses incorporating missing values, which decreased slightly to 1897.7 units (IQR 1599.8) once those cases were removed. TMPRSS2 presented a median expression of 113 units with an IQR of 54.4. With respect to sensory symptoms, alterations in taste perception were absent in 63.5% of the participants (*n* = 66) and were reported by 36.5% (*n* = 38). Similarly, 62.5% of the participants (*n* = 65) denied olfactory symptoms, whereas 37.5% (*n* = 39) acknowledged experiencing smell dysfunction.

Table 2 shows the results of binary logistic regression assessing the associations between receptor counts (furin, ACE2, NRP1, and TMPRSS2) and the presence of olfactory dysfunction, both unadjusted and adjusted for age, sex, and race. In the adjusted model, each 100-unit increase in furin was associated with an OR = 1.05 greater risk of olfactory dysfunction in patients with COVID-19 in the imputed dataset, although this result was not statistically significant (*p* = 0.821). No statistically significant association was observed between ACE2 expression and olfactory dysfunction after adjustment (*p* = 0.320, OR = 1.02). Similarly, no association was found for NRP1 (*p* = 0.772, OR = 1.00). TMPRSS2 levels were also not significantly associated with severity after adjustment (*p* = 0.338, OR = 0.78).

Table 3 further reports the binary logistic regression results for evaluating the association between receptor status and olfactory dysfunction in patients. As in the previous analysis, the receptors studied were furin, ACE2, NRP1, and TMPRSS2, and outcomes were compared between patients with and without olfactory dysfunction. In the adjusted model, the associations between furin levels and olfactory dysfunction lost statistical relevance (medium level: *p* = 0.092, OR = 3.16; high level: *p* = 0.166, OR = 2.30). We observed the same results for ACE2 levels (medium: *p* = 0.128, OR = 2.62; high: *p* = 0.296, OR = 1.88). NRP1 also showed the same results (medium: *p* = 0.116, OR = 0.38; high: *p* = 0.997, OR= 1.00) but a low correlation at the medium level. Neither medium nor high TMPRSS2 expression was significantly associated with olfactory dysfunction after adjustment (medium: *p* = 0.702, OR = 1.24; high: *p* = 0.158, OR = 0.41).

Table 4 summarizes the findings of the binary logistic regression analysis conducted to examine the relationship between receptor expression levels (furin, ACE2, NRP1, and TMPRSS2) and the presence of dysgeusia in individuals diagnosed with COVID-19. Two analytical approaches were reported: a crude model and a multivariable model incorporating adjustments for age, sex, and race. After controlling for age, sex, and race, no statistically significant relationships were identified between furin expression levels and dysgeusia (OR = 1.23; *p* = 0.319). No statistically significant association was detected between ACE2 expression and dysgeusia (OR = 1.04; *p* = 0.115). Similarly, the adjusted analysis did not reveal a statistically significant association between NRP1 expression levels and dysgeusia (OR = 1.01; *p* = 0.195). No statistically meaningful association was observed when TMPRSS2 was examined in relation to the presence of dysgeusia (OR= 1.17; *p* = 0.506).

Table 5 shows the results of a binary logistic regression analysis conducted to investigate the relationships between the expression levels of furin, ACE2, NRP1, and TMPRSS2 and the presence of dysgeusia in patients with severe COVID-19. Two analytical models were provided: an unadjusted model and a multivariable model controlling for age, sex, and race. Intermediate and high furin expression levels were associated with an increased likelihood of dysgeusia (ORs = 2.56 and 2.83, respectively); however, these relationships did not reach statistical significance (intermediate furin expression, *p* = 0.153; high furin expression, *p*= 0.072). Moderate ACE2 expression was associated with a greater estimated risk of dysgeusia (OR = 2.32), although this effect was not statistically significant (*p* = 0.169). Similarly, elevated ACE2 levels were not significantly associated with dysgeusia (*p* = 0.156, OR = 2.29). Intermediate NRP1 expression was associated with a reduced estimated risk of dysgeusia (OR = 0.30), with a *p* value approaching the threshold for statistical significance (*p* = 0.058). High NRP1 expression was not significantly associated with dysgeusia (*p* = 0.451, OR = 1.52). After adjusting for covariates, no statistically significant associations were identified between TMPRSS2 expression levels and the presence of dysgeusia, either at intermediate (*p* = 0.479, OR = 1.47) or high expression levels (*p* = 0.321, OR = 0.55).

## 4. Discussion

Despite previous experimental evidence suggesting a role for these receptors in the pathophysiology of infection, our binary logistic regression analyses did not identify significant associations between receptor expression levels and the development of olfactory dysfunction, either in unadjusted models or in models adjusted for age, sex, and race, using both complete-case analyses and datasets with imputed missing values. However, a positive association was observed between furin and ACE2 levels and the presence of this symptom, although the results were not significant. These results should not be interpreted as contradictory to previous studies reporting high ACE2 expression in the olfactory neuroepithelium, which facilitates SARS-CoV-2 entry into cells of the olfactory system and contributes to sensory dysfunction in COVID-19 (e.g., Chen et al. [23]; Doty et al. [24]); rather, the two sets of findings address different biological compartments and timepoints, as the present study did not specifically examine olfactory epithelial tissue or sustentacular cells. Similarly, previous research has shown that ACE2 and TMPRSS2 are predominantly expressed in nonneuronal cells of the olfactory epithelium, such as sustentacular and glial cells, suggesting that olfactory damage is mediated mainly by inflammatory and indirect mechanisms rather than by primary neuronal injury [12,25]. This pathophysiological model could explain the generally transient nature of olfactory dysfunction associated with SARS-CoV-2 infection [1,12,14,26,27].

The stratified analysis by tertiles reinforced these findings, as no significant associations were observed between low, intermediate, or high levels of NRP1 and TMPRSS2 and olfactory dysfunction. However, a nonsignificant positive trend was observed between higher furin and ACE2 levels and an increased risk of olfactory dysfunction. While these results did not reach statistical significance, they could indicate an underlying biological effect that was not detected because of limitations inherent in the sample size or the complexity of the molecular interactions involved.

Although furin has been less studied in the context of COVID-19-associated olfactory dysfunction, it plays a key role in activating the spike protein through proteolytic cleavage, facilitating the interaction between ACE2 and TMPRSS2 [23,25,28]. Its expression in supporting cells and olfactory neurons could contribute to local inflammatory processes and epithelial damage, which would explain the observed tendency toward a higher risk of olfactory alterations in individuals with elevated levels of this receptor.

For NRP1, even if no direct association with olfactory dysfunction was identified, a relevant finding was observed in the analysis of disease severity. Intermediate levels of NRP1 were significantly associated with decreased COVID-19 severity in the adjusted models, suggesting a possible modulatory or protective role for this receptor. Given that mild forms of the disease are more frequently associated with the presence of olfactory dysfunction [2,25], this result indicates an indirect relationship between NRP1, the clinical course of infection, and the onset of sensory symptoms. The scarcity of studies addressing this topic highlights the need for further research on this receptor.

The absence of significant associations in our analysis suggests that the expression of these receptors, assessed at a single time point, may not adequately reflect the dynamic processes that occur during the different phases of infection. In this context, temporal variations in receptor expression, together with the local immune response, viral load, and inflammatory microenvironment, likely play decisive roles in the development and resolution of olfactory dysfunction. Therefore, we believe that olfactory alterations should be considered multifactorial and dynamic phenomena rather than direct and linear consequences of the expression of a single receptor.

Similarly, we did not reveal significant associations between the levels of furin, ACE2, NRP1, or TMPRSS2 and taste disturbance. These results contrast with those of studies that reported high expression of ACE2, TMPRSS2, and furin in the oral mucosal epithelium and taste cells, suggesting a possible mechanism of viral entry and direct sensory damage [29,30,31,32,33,34,35].

On the other hand, the analysis of expression levels revealed a consistent, albeit nonsignificant, trend toward a greater probability of dysgeusia in individuals with elevated furin levels, especially in the unadjusted models. This finding suggests the possible involvement of furin in the pathogenesis of COVID-19-associated dysgeusia, although its effect appears to be influenced by confounding factors such as age, sex, and race. In contrast, ACE2 showed no conclusive associations despite a nonsignificant increase in risk at medium and high levels, whereas NRP1 and TMPRSS2 showed nonsignificant trends toward a possible protective effect at intermediate values.

Notably, dysgeusia is a clinically complex and often poorly characterized symptom, as it is often confused with other symptoms of olfactory dysfunction. This diagnostic difficulty may have influenced the classification of cases and, consequently, the ability of the study to detect significant associations.

Taken together, our findings indicate that persistent expression levels of SARS-CoV-2 entry-related genes (furin, ACE2, NRP1, and TMPRSS2) in nasal or oropharyngeal swab samples during the post-acute phase are not significantly associated with olfactorydysfunction or dysgeusia under the specific conditions studied. These results should not be interpreted as excluding a role for these receptors in the pathogenesis of sensory disturbances but rather as reflecting the limitations inherent to late post-infection, cross-sectional assessment.

Olfactory dysfunction and dysgeusia are more likely to arise from complex, multifactorial processes, involving interactions between multiple viral entry pathways, inflammatory responses, host susceptibility factors, and early epithelial injury during the acute phase of infection. In this context, future studies incorporating longitudinal designs, acute-phase sampling, standardized symptom phenotyping, and multivariate or receptor–receptor interaction models will be essential to further elucidate the mechanisms underlying COVID-19-related sensory dysfunction and to inform the development of targeted therapeutic strategies.

### Methodological Considerations

This study has several methodological limitations that warrant consideration. First, the relatively small sample size, partly attributable to participant refusal and mobility restrictions enforced during the state of alarm declared by the Spanish government, may have limited the statistical power to detect modest associations. In addition, the study population was restricted to individuals from the Region of Murcia, which may constrain the external validity and generalizability of the findings to other geographic or epidemiological settings. Second, gene expressions were assessed using nasal and oropharyngeal swab-derived material, which predominantly reflects respiratory epithelial cells, and cellular composition was not verified using cell-type-specific markers. Consequently, the results cannot be directly extrapolated to the olfactory epithelium, including sustentacular cells or Bowman gland cells, which are known to play a central role in COVID-19-related olfactory dysfunction. Third, samples were obtained approximately 75 days after COVID-19 diagnosis, corresponding to a post-acute phase in which viral clearance has occurred and epithelial regeneration may be ongoing or completed. Receptor expression measured at this timepoint is therefore unlikely to reflect biological mechanisms driving the acute onset of olfactory dysfunction or dysgeusia and precludes temporal or causal inference. Fourth, cutoff values for nasal receptor expression were defined by the authors due to the absence of previously established standardized reference ranges, which may have introduced classification uncertainty. Finally, olfactory dysfunction and dysgeusia were assessed using self-reported measures, and the accurate identification of dysgeusia remains challenging, as it represents a subjective sensory alteration that is frequently confounded with olfactory dysfunction. This potential nondifferential misclassification may have attenuated true associations.

In contrast, the main strengths of this investigation include its multicenter analytical design, which enhances the methodological rigor and representativeness of the results, as well as the inclusion of PCR-confirmed cases, ensuring diagnostic accuracy in the evaluation of disease-related associations. Moreover, the direct quantification of nasal receptor expression provides objective and novel evidence regarding the potential contribution of nasal receptor expression to COVID-19 severity. The application of statistical models adjusted for relevant confounders, including age, sex, and race, further strengthens the robustness of the findings. Finally, the generation of original data from the Region of Murcia within the context of the COVID-19 pandemic adds clinically relevant and applicable evidence to the literature.

## 5. Conclusions

In this cross-sectional study, we did not identify statistically significant associations between nasal expression levels of SARS-CoV-2 entry-related genes (ACE2, TMPRSS2, furin, and NRP1) and self-reported olfactory dysfunction or dysgeusia in SARS-CoV-2 infection. These findings should be interpreted with caution and viewed as exploratory, given the limited sample size, the post-acute timing of sample collection, and the use of subjective, binary outcome measures. The absence of statistically significant associations under these specific conditions does not preclude a causal role of these receptors in the pathophysiology of COVID-19-related chemosensory dysfunction during the acute phase of infection. Rather, our results highlight the complex and multifactorial nature of olfactory dysfunction and dysgeusia, likely involving early epithelial injury, viral load, inflammatory responses, temporal variation in receptor expression, and host susceptibility factors. These observations underscore the need for longitudinal, adequately powered studies incorporating standardized and objective sensory assessments, acute-phase sampling, and multivariate analytical approaches to more precisely define the contribution of nasal entry receptors to persistent olfactory and gustatory symptoms following SARS-CoV-2 infection.

## Figures and Tables

**Table 1:** Descriptive data of the study patients.

Variables		Database with Missing Data	Imputed Database
Age	Median (IQR)	58.5 (16.2)	58.5 (16.2)
Sex	Male (%)	69 (66.3)	69 (66.3)
	Female	35 (33.7)	35 (33.7)
Race/Ethnicity	Caucasian (%)	85 (81.7)	85
	Latino (%)	19 (18.3)	19 (18.3)
Furin (units)	Median (IQR)	212.3 (105.1)	213.0 (103.8)
ACE2 (units)	Median (IQR)	2098.3 (1271.3)	2098.3 (1311.7)
NRP1 (units)	Median (IQR)	1904.3 (1627.6)	1897.7 (1599.8)
TMPRSS2 (units)	Median (IQR)	113.0 (54.4)	113.0 (54.0)
Olfactory dysfunction	No	63 (61.8)	65 (62.5)
	Yes (%)	39 (38.2)	39 (37.5)
Dysgeusia	No	66 (63.5)	66 (63.5)
	Yes (%)	38 (36.5)	38 (36.5)

ACE2, angiotensin-converting enzyme 2; NRP1, neuropilin-1; IQR, interquartile range; TMPRSS2, transmembrane serine protease 2.

**Table 2:** Binary logistic regression analysis evaluating the association between the number of receptors and olfactory dysfunction in patients.

	Database with Missing Data			Imputed Database		
	Olfactory Dysfunction			Olfactory Dysfunction		
Predictor	OR	95% CI	*p*	OR	95% CI	*p*
Unadjusted model						
Furin (per 100 units)	1.04	0.71 to 1.52	0.835	1.04	0.72 to 1.51	0.827
Adjusted model ^†^						
Furin (per 100 units)	1.05	0.67 to 1.66	0.822	1.05	0.68 to 1.63	0.821
Unadjusted model						
ACE2 (per 100 units)	1.00	0.96 to 1.04	0.853	1.01	0.97 to 1.05	0.664
Adjusted model ^†^						
ACE2 (per 100 units)	1.02	0.97 to 1.06	0.446	1.02	0.98 to 1.07	0.320
Unadjusted model						
NRP1 (per 100 units)	1.00	0.98 to 1.02	0.941	1.0	0.98 to 1.02	0.789
Adjusted model ^†^						
NRP1 (per 100 units)	1.00	0.98 to 1.02	0.861	1.0	0.98 to 1.03	0.772
Unadjusted model						
TMPRSS2 (per 100 units)	1.04	0.68 to 1.60	0.852	1.04	0.68 to 1.59	0.863
Adjusted model ^†^						
TMPRSS2 (per 100 units)	0.80	0.48 to 1.32	0.378	0.78	0.47 to 1.29	0.338

^†^ Adjusted for age, sex, and race/ethnicity; ACE2, angiotensin-converting enzyme 2; CI, confidence interval; NRP1, neuropilin-1; OR, odds ratio; *p*, statistical significance; TMPRSS2, transmembrane serine protease 2.

**Table 3:** Binary logistic regression analysis evaluating the association between receptor number status and olfactory dysfunction in patients.

	Database with Missing Data			Imputed Database		
	Olfactory Dysfunction			Olfactory Dysfunction		
Predictor	OR	95% CI	*p*	OR	95% CI	*p*
Unadjusted model						
Low furin level	Ref.			Ref.		
Medium furin level	1.38	0.49 to 3.87	0.541	1.42	0.52 to 3.87	0.496
High furin level	2.04	0.75 to 5.58	0.162	1.99	0.75 to 5.25	0.164
Adjusted model ^†^						
Low furin level	Ref.			Ref.		
Medium furin level	3.35	0.82 to 13.64	0.091	3.16	0.83 to 12.00	0.092
High furin level	2.36	0.68 to 8.17	0.176	2.30	0.71 to 7.44	0.166
Unadjusted model						
Low ACE2 level	Ref.			Ref.		
Medium ACE2 level	1.20	0.44 to 3.25	0.724	1.25	0.46 to 3.35	0.663
High ACE2 level	0.95	0.35 to 2.59	0.927	0.82	0.31 to 2.16	0.687
Adjusted model ^†^						
Low ACE2 level	Ref.			Ref.		
Medium ACE2 level	2.86	0.80 to 10.26	0.107	2.62	0.76 to 9.09	0.128
High ACE2 level	1.51	0.44 to 5.12	0.513	1.88	0.58 to 6.15	0.296
Unadjusted model						
Low NRP1 level	Ref.			Ref.		
Medium level NRP1	0.54	0.19 to 1.53	0.249	0.46	0.17 to 1.26	0.130
High NRP1 level	0.53	0.20 to 1.44	0.942	1.02	0.38 to 2.68	0.976
Adjusted model ^†^						
Low NRP1 level	Ref.			Ref.		
Medium level NRP1	0.49	0.14 to 1.68	0.254	0.38	0.12 to 1.27	0.116
High NRP1 level	1.03	0.32 to 3.29	0.965	1.0	0.33 to 3.05	0.997
Unadjusted model						
Low TMPRSS2 level	Ref.			Ref.		
Medium level TMPRSS2	1.36	0.51 to 3.63	0.543	1.33	0.51 to 3.46	0.555
High level TMPRSS2	0.53	0.19 to 1.50	0.232	0.52	0.19 to 1.44	0.207
Adjusted model ^†^						
Low TMPRSS2 level	Ref.			Ref.		
Medium level TMPRSS2	1.37	0.45 to 4.19	0.583	1.24	0.41 to 3.72	0.702
High level TMPRSS2	0.44	0.13 to 1.52	0.194	0.41	0.12 to 1.42	0.158

^†^ Adjusted for age, sex, and race/ethnicity ACE2, angiotensin-converting enzyme 2; CI, confidence interval; NRP1, neuropilin-1; OR, odds ratio; *p*, statistical significance; Ref., reference; TMPRSS2, transmembrane serine protease 2.

**Table 4:** Binary logistic regression analysis evaluating the association between the number of receptors and dysgeusia in patients.

	Database with Missing Data			Imputed Database		
	Dysgeusia			Dysgeusia		
Predictor	OR	95% CI	*p*	OR	95% CI	*p*
Unadjusted model						
Furin (per 100 units)	1.19	0.82 to 1.73	0.371	1.20	0.83 to 1.75	0.339
Adjusted model ^†^						
Furin (per 100 units)	1.22	0.81 to 1.84	0.337	1.23	0.82 to 1.83	0.319
Unadjusted model						
ACE2 (per 100 units)	1.02	0.98 to 1.06	0.335	1.02	0.98 to 1.06	0.263
Adjusted model ^†^						
ACE2 (per 100 units)	1.03	0.99 to 1.08	0.159	1.04	0.99 to 1.08	0.115
Unadjusted model						
NRP1 (per 100 units)	1.01	0.99 to 1.03	0.262	1.01	0.99 to 1.03	0.210
Adjusted model ^†^						
NRP1 (per 100 units)	1.01	0.99 to 1.04	0.215	1.01	0.99 to 1.04	0.195
Unadjusted model						
TMPRSS2 (per 100 units)	1.34	0.87 to 2.07	0.186	1.30	0.85 to 2.00	0.228
Adjusted model ^†^						
TMPRSS2 (per 100 units)	1.20	0.75 to 1.92	0.44	1.17	0.74 to 1.86	0.506

^†^ Adjusted for age, sex, and race/ethnicity ACE2, angiotensin-converting enzyme 2 CI, confidence interval; NRP1, neuropilin-1; OR, odds ratio; *p*, statistical significance; TMPRSS2, transmembrane serine protease 2.

**Table 5:** Binary logistic regression analysis evaluating the association between the status of receptors and dysgeusia in patients.

	Database with Missing Data			Imputed Database		
	Dysgeusia			Dysgeusia		
Predictor	OR	95% CI	*p*	OR	95% CI	*p*
Unadjusted model						
Low furin level	Ref.			Ref.		
Medium furin level	1.45	0.51 to 4.18	0.487	1.41	0.50 to 3.97	0.510
High furin level	2.62	0.95 to 7.21	0.062	2.55	0.95 to 6.82	0.062
Adjusted model ^†^						
Low furin level	Ref.			Ref.		
Medium furin level	2.72	0.71 to 10.47	0.144	2.56	0.70 to 9.30	0.153
High furin level	2.89	0.88 to 9.45	0.080	2.83	0.91 to 8.78	0.072
Unadjusted model						
Low ACE2 level	Ref.			Ref.		
Medium ACE2 level	1.31	0.48 to 3.60	0.602	1.25	0.46 to 3.42	0.668
High ACE2 level	1.35	0.50 to 3.65	0.553	1.56	0.59 to 4.13	0.372
Adjusted model ^†^						
Low ACE2 level	Ref.			Ref.		
Medium ACE2 level	2.56	0.76 to 8.62	0.128	2.32	0.70 to 7.69	0.169
High ACE2 level	1.92	0.60 to 6.21	0.274	2.29	0.73 to 7.21	0.156
Unadjusted model						
Low NRP1 level	Ref.			Ref.		
Medium level NRP1	0.49	0.17 to 1.43	0.190	0.39	0.14 to 1.12	0.081
High NRP1 level	1.49	0.55 to 4.03	0.432	1.46	0.55 to 3.87	0.445
Adjusted model ^†^						
Low level NRP1	Ref.			Ref.		
Medium level NRP1	0.39	0.11 to 1.38	0.144	0.30	0.09 to 1.04	0.058
High NRP1 level	1.66	0.53 to 5.15	0.381	1.52	0.51 to 4.55	0.451
Unadjusted model						
Low TMPRSS2 level	Ref.			Ref.		
Intermediate TMPRSS2	1.53	0.57 to 4.08	0.398	1.50	0.58 to 3.93	0.405
High level TMPRSS2	0.63	0.22 to 1.79	0.390	0.59	0.21 to 1.63	0.306
Adjusted model ^†^						
Low TMPRSS2 level	Ref.			Ref.		
Medium level TMPRSS2	1.61	0.55 to 4.73	0.388	1.47	0.51 to 4.23	0.479
High level TMPRSS2	0.60	0.18 to 1.98	0.406	0.55	0.17 to 1.79	0.321

^†^ Adjusted for age, sex, and race/ethnicity ACE2, angiotensin-converting enzyme 2; CI, confidence interval; NRP1, neuropilin-1; OR, odds ratio; *p*, statistical significance; Ref., reference; TMPRSS2, transmembrane serine protease 2.

## Data Availability

The datasets used and analyzed in the current study are available from the corresponding author upon reasonable request.

## Abbreviations

The following abbreviations are used in this manuscript:

COVID-19	Coronavirus Disease 2019
SARS-CoV-2	Severe Acute Respiratory Syndrome Coronavirus 2
ACE2	Angiotensin-Converting Enzyme 2
TMPRSS2	Transmembrane Protease, Serine 2
NRP-1	Neuropilin 1
PCR	Polymerase Chain Reaction

## References

[1] Hajikhani B., Calcagno T., Nasiri M.J., Jamshidi P., Dadashi M., Goudarzi M., Eshraghi A.A., Mirsaeidi M., FACS (2020). Olfactory and gustatory dysfunction in COVID-19 patients: A meta-analysis study. Physiol. Rep..

[2] Lechien J.R., Chiesa-Estomba C.M., De Siati D.R., Horoi M., Le Bon S.D., Rodriguez A., Dequanter D., Blecic S., Afia F.E., Distinguin L. (2020). Olfactory and gustatory dysfunctions as a clinical presentation of mild-to-moderate forms of the coronavirus disease (COVID-19): A multicenter European study. Eur. Arch. Oto-Rhino-Laryngol..

[3] Lechien J.R., Ducarme M., Place S., Chiesa-Estomba C.M., Khalife M., De Riu G., Vaira L.A., de Terwangne C., Machayekhi S., Marchant A. (2020). Objective Olfactory Findings in Hospitalized Severe COVID-19 Patients. Pathogens.

[4] Santamaría-Gadea A., Izquierdo-Domínguez A., Mullol J., Alobid I. (2022). Disfunción Olfatoria en la COVID-19 Persistente.

[5] Rogn Å., Jensen J.L., Iversen P.O., Singh P.B. (2024). Post-COVID-19 patients suffer from chemosensory, trigeminal, and salivary dysfunctions. Sci. Rep..

[6] von Bartheld C.S., Hagen M.M., Butowt R. (2020). Prevalence of Chemosensory Dysfunction in COVID-19 Patients: A Systematic Review and Meta-analysis Reveals Significant Ethnic Differences. ACS Chem. Neurosci..

[7] Mutiawati E., Fahriani M., Mamada S.S., Fajar J.K., Frediansyah A., Maliga H.A., Ilmawan M., Bin Emran T., Ophinni Y., Ichsan I. (2021). Anosmia and dysgeusia in SARS-CoV-2 infection: Incidence and effects on COVID-19 severity and mortality, and the possible pathobiology mechanisms-a systematic review and meta-analysis. F1000 Res..

[8] Butowt R., Bilinska K., von Bartheld C.S. (2023). Olfactory dysfunction in COVID-19: New insights into the underlying mechanisms. Trends Neurosci..

[9] Mayi B.S., Leibowitz J.A., Woods A.T., Ammon K.A., Liu A.E., Raja A. (2021). The role of Neuropilin-1 in COVID-19. PLoS Pathog..

[10] Daly J.L., Simonetti B., Klein K., Chen K.E., Williamson M.K., Antón-Plágaro C., Shoemark D.K., Simón-Gracia L., Bauer M., Hollandi R. (2020). Neuropilin-1 is a host factor for SARS-CoV-2 infection. Science.

[11] Izquierdo-Domínguez A., Rojas-Lechuga M.J., Mullol J., Alobid I. (2020). Pérdida del sentido del olfato durante la pandemia COVID-19. Med. Clínica.

[12] Lechien J.R., Radulesco T., Calvo-Henriquez C., Chiesa-Estomba C.M., Hans S., Barillari M.R., Cammaroto G., Descamps G., Hsieh J., Vaira L. (2020). ACE2 & TMPRSS2 Expressions in Head & Neck Tissues: A Systematic Review. Head Neck Pathol..

[13] Lechien J.R., Chiesa-Estomba C.M., Hans S., Barillari M.R., Jouffe L., Saussez S. (2020). Loss of Smell and Taste in 2013 European Patients with Mild to Moderate COVID-19. Ann. Intern. Med..

[14] Bilinska K., Jakubowska P., Von Bartheld C.S., Butowt R. (2020). Expression of the SARS-CoV-2 Entry Proteins, ACE2 and TMPRSS2, in Cells of the Olfactory Epithelium: Identification of Cell Types and Trends with Age. ACS Chem. Neurosci..

[15] Divani A.A., Andalib S., Di Napoli M., Lattanzi S., Hussain M.S., Biller J., McCullough L.D., Azarpazhooh M.R., Seletska A., Mayer S.A. (2020). Coronavirus Disease 2019 and Stroke: Clinical Manifestations and Pathophysiological Insights. J. Stroke Cerebrovasc. Dis..

[16] Hopkins C., Surda P., Whitehead E., Kumar B.N. (2020). Early recovery following new onset anosmia during the COVID-19 pandemic-an observational cohort study. J. Otolaryngol.-Head Neck Surg..

[17] Politi L.S., Salsano E., Grimaldi M. (2020). Magnetic Resonance Imaging Alteration of the Brain in a Patient With Coronavirus Disease 2019 (COVID-19) and Anosmia. JAMA Neurol..

[18] Toledano A., Borromeo S., Luna G., Molina E., Solana A.B., García-Polo P., Hernandez J.A., Alvarez-linera J. (2012). Estudio objetivo del olfato mediante resonancia magnética funcional. Acta Otorrinolaringológica Esp..

[19] Kasiri H., Rouhani N., Salehifar E., Ghazaeian M., Fallah S. (2021). Mometasone furoate nasal spray in the treatment of patients with COVID-19 olfactory dysfunction: A randomized, double blind clinical trial. Int. Immunopharmacol..

[20] Sociedad Iberoamericana de Información Científica Corticoides Intranasales Para la Recuperación del Sentido del Olfato en Pacientes Con COVID-19. https://www.siicsalud.com/dato/resiiccompleto.php/165751.

[21] Pfaar O., Klimek L., Jutel M., Akdis C.A., Bousquet J., Breiteneder H., Chinthrajah S., Diamant Z., Eiwegger T., Fokkens W.J. (2021). COVID-19 pandemic: Practical considerations on the organization of an allergy clinic-An EAACI/ARIA Position Paper. Allergy.

[22] Bousquet J., Akdis C.A., Jutel M., Bachert C., Klimek L., Agache I., Ansotegui I.J., Bedbrook A., Bosnic-Anticevich S., Canonica G.W. (2020). Intranasal corticosteroids in allergic rhinitis in COVID-19 infected patients: An ARIA-EAACI statement. Allergy.

[23] Chen M., Shen W., Rowan N.R., Kulaga H., Hillel A., Ramanathan M., Lane A.P. (2020). Elevated ACE2 expression in the olfactory neuroepithelium: Implications for anosmia and upper respiratory SARS-CoV-2 entry and replication. Eur. Respir. J..

[24] Doty R.L. (2022). Olfactory dysfunction in COVID-19: Pathology and long-term implications for brain health. Trends Mol. Med..

[25] Brann D.H., Tsukahara T., Weinreb C., Lipovsek M., Van den Berge K., Gong B., Chance R., Macaulay I.C., Chou H.-J., Fletcher R.B. (2020). Non-neuronal expression of SARS-CoV-2 entry genes in the olfactory system suggests mechanisms underlying COVID-19-associated anosmia. Sci. Adv..

[26] Lima M.A., Silva M.T.T., Oliveira R.V., Soares C.N., Takano C.L., Azevedo A.E., Moraes R.L., Rezende R.B., Chagas I.T., Espíndola O. (2020). Smell dysfunction in COVID-19 patients: More than a yes-no question. J. Neurol. Sci..

[27] Samaranayake L.P., Fakhruddin K.S., Panduwawala C. (2020). Sudden onset, acute loss of taste and smell in coronavirus disease 2019 (COVID-19): A systematic review. Acta Odontol. Scand..

[28] Osman E.E.A., Rehemtulla A., Neamati N. (2022). Why All the Fury over Furin?. J. Med. Chem..

[29] Zang R., Gomez Castro M.F., McCune B.T., Zeng Q., Rothlauf P.W., Sonnek N.M., Liu Z., Brulois K.F., Wang X., Greenberg H.B. (2020). TMPRSS2 and TMPRSS4 promote SARS-CoV-2 infection of human small intestinal enterocytes. Sci. Immunol..

[30] Bourgonje A.R., Abdulle A.E., Timens W., Hillebrands J., Navis G.J., Gordijn S.J., Bolling M.C., Dijkstra G., Voors A.A., Osterhaus A.D. (2020). Angiotensin-converting enzyme 2 (ACE2), SARS-CoV-2 and the pathophysiology of coronavirus disease 2019 (COVID-19). J. Pathol..

[31] Sungnak W., Huang N., Bécavin C., Berg M. (2020). HCA Lung Biological Network. SARS-CoV-2 Entry Genes Are Most Highly Expressed in Nasal Goblet and Ciliated Cells within Human Airways. arXiv.

[32] Xu H., Zhong L., Deng J., Peng J., Dan H., Zeng X., Li T., Chen Q. (2020). High expression of ACE2 receptor of 2019-nCoV on the epithelial cells of oral mucosa. Int. J. Oral Sci..

[33] Doyle M.E., Appleton A., Liu Q.R., Yao Q., Mazucanti C.H., Egan J.M. (2021). Human Taste Cells Express ACE2: A Portal for SARS-CoV-2 Infection. bioRxiv.

[34] Hoffmann M., Kleine-Weber H., Schroeder S., Krüger N., Herrler T., Erichsen S., Schiergens T.S., Herrler G., Wu N.-H., Nitsche A. (2020). SARS-CoV-2 Cell Entry Depends on ACE2 and TMPRSS2 and Is Blocked by a Clinically Proven Protease Inhibitor. Cell.

[35] Heurich A., Hofmann-Winkler H., Gierer S., Liepold T., Jahn O., Pöhlmann S. (2014). TMPRSS2 and ADAM17 cleave ACE2 differentially and only proteolysis by TMPRSS2 augments entry driven by the severe acute respiratory syndrome coronavirus spike protein. J. Virol..

